# Where the wolf roams: ecological preferences and wild prey association in a changing Mediterranean landscape

**DOI:** 10.1186/s12983-026-00598-2

**Published:** 2026-02-05

**Authors:** Maria Buglione, Domenico Fulgione, Tiziano Trasmondo, Benedetta De Francesco, Gabriele de Filippo, Eleonora Rivieccio

**Affiliations:** 1 Department of Biology, University of Naples Federico II, Via Cupa Nuova Cinthia 26, 80126 Naples, Italy; 2 Department of Humanities, University of Naples Federico II, Via Porta di Massa 1, 80133 Naples, Italy; 3 Istituto di Gestione della Fauna (IGF), Via M da Caravaggio 143, 80126 Naples, Italy

**Keywords:** Apennine mountain landscape, *Canis lupus*, National Park management, Prey–predator interaction, Wild ungulates

## Abstract

The wolf (*Canis lupus*) is a species of great conservation interest and has recently highlighted management challenges throughout Europe. Over the past 50 years, the establishment of National Parks, forest cover changes as well as the expansion of wild ungulates seem to have played a crucial role in shaping the demographic trend of the wolf. We investigated prey–predator associations in the Cilento, Vallo di Diano e Alburni National Park (Southern Italy), one of the most ecologically significant areas contributing to the recovery of wolf in Southern Apennine. During the wolf bottleneck in the 70’s, the protected area hosted only a few wolves and wild prey were scarce, while nowadays the wolf population has exceeded two hundred individuals, the wild boar number has increased significantly, and both roe deer and red deer are steadily rising following reintroduction actions. We aim to examine the relationship between the wolf population and its three primary wild prey considering the environmental changes that have occurred over the past few decades to underpin the current and future complex ecological relationships in Mediterranean forest ecosystems. Overall, our study lays the groundwork for future management strategies aimed at reconciling large carnivore conservation with sustainable forest use and human–wildlife coexistence.

**Supplementary Information:**

The online version contains supplementary material available at 10.1186/s12983-026-00598-2.

## Background

**Fig. 1:**
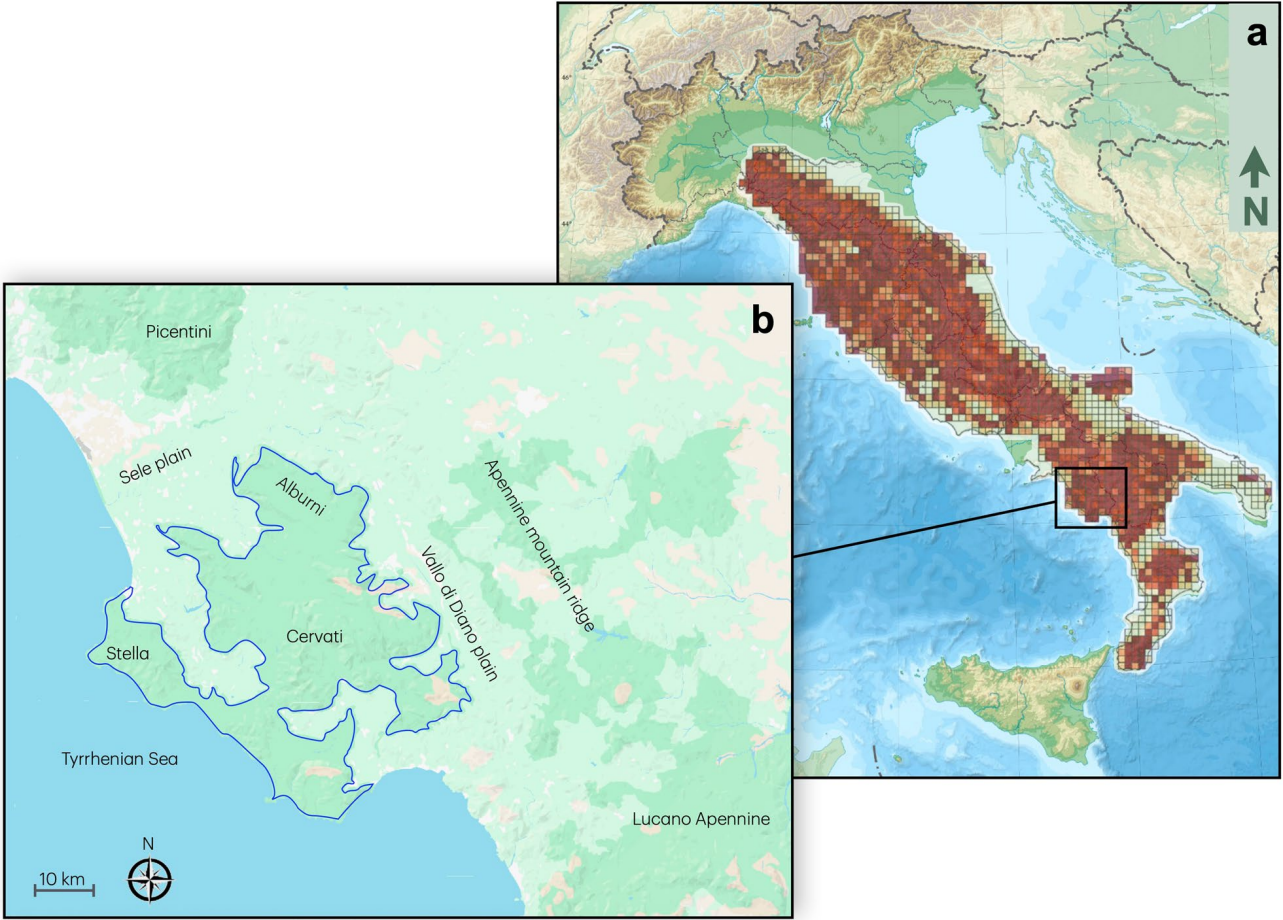
Study area. **a** In shades of red, cell-specific wolf occupancy probability in Italy from [30] (modified); **b** In dark green delimited by the blue line, the Cilento, Vallo di Diano e Alburni National Park; the names of the sites mentioned in the main text were indicated

The wolf (*Canis lupus*) is a keystone predator that has played a crucial role in shaping the ecological dynamics and biodiversity across the Italian Peninsula [1–3]. Following a dramatic population decline during the 19th and early twentieth centuries, when fewer than 100 individuals survived primarily in the Central and Southern Apennines (from Abruzzo to Calabria) [4], the species has markedly recovered thanks to legal protection, promoting awareness and positive perception, habitat restoration, and increase in wild ungulates [5–11]. In 2020–2021, it was reported Italy hosted approximately 3,307 wolves, including 2,020–2,645 wolves in the Apennines and 822–1099 in the Alps [12] (Fig. 1a).

Recently, the status of protection of wolf in Europe was formally downgraded from “strictly protected” to “protected” under the Bern Convention and EU Habitats Directive highlighting the urgency of revising and implementing effective management measures for this predator. These strategies should be informed by a detailed understanding of knowledge of the species and its ecological demands, and must prioritize appropriate stewardship of both the territory, especially within protected areas, and the wolf’s trophic niche.

In this contribution, we focused on the wolf population in the Cilento, Vallo di Diano e Alburni National Park (PNCVDA), one of the most ecologically significant areas contributing to the recovery of wolf. Located in Campania (Southern Apennines, Italy), this protected area offers a suitable refuge for wolves and a recovering prey base, encompassing a diverse mosaic of forests, meadows, and rugged mountains (Fig. 1b).

In Campania, as in the rest of the Italian peninsula, wolf population underwent a significant decline sharply after World War II, persisting during1970s only in a few core areas (Matese, Picentini, and Cilento) connected by dispersing individuals. Subsequently, following the national trend, the population recovered, although regional information remained scarce and largely anecdotal until 1990s. Early assessment (1985–1999) and Natura 2000 reports indicated presence of wolves in mountainous areas while later records (2003) confirmed a broader distribution throughout the Apennines in Campania, although they still highlighted need for updated data. Thus, the apparent population increase may reflect improved data collection as well as genuine expansion. In 2017, more recent surveys using genetic and standardized methods, identified stable packs in the Alburni Mountains and the Mount Cervati with at least 3 packs (4–7 individuals each), likely representing source populations [13]. Overall, it was reported a potential wolf population of around 62 individuals in Cilento core area [14]. Finally, a density of about 2.9 individuals per 100 km^2^ was reported in parts of Benevento and Avellino in 2022, but no genetically data are yet available for the PNCVDA [12].

During the nineteenth and twentieth centuries, also large wild herbivores and scavengers mammals in the Southern Italy underwent severe population declines or local extinctions, due to overhunting, agricultural expansion, and deforestation [15, 16]. In the PNCVDA, while wild boar (*Sus scrofa*) persists at low densities, both roe deer (*Capreolus capreolus*) and red deer (*Cervus elaphus*) disappeared by the early 1950s [17–19]. Subsequently, increasing in forest cover expansion and the land-use modification across Apennines [20, 21] created favorable conditions for the recolonization and reintroduction of ungulates. Between 2003–2006, Park Authority reintroduced 37 roe deer and 35 red deer on Mount Cervati (Fig. 1b) with recent surveys estimating mean density of 1.57 ind/km^2^ ± 0.37 and 0.54 ind/km^2^ ± 0.25 for roe deer and red deer, respectively [19].

Wild boar was historically present in the PNCVDA [22, 23], where current densities often exceed 10 ind/km^2^, well above the ecological threshold of 2–3 ind/km^2^ bearable in Mediterranean agroecosystems [24, 25]. The species now invaded all types of habitats, including those modified by humans [26, 27].

As a final remark, due to the sharp landscape remodeling, ecological interactions in Mediterranean habitats (particularly long-disrupted predator–prey dynamics), are being gradually restructured, influencing wild herbivore population sizes, predator population growth, and ultimately overall ecosystem biodiversity.

Here, we aimed to examine the relationship between the wolf population and its three primary wild prey (roe deer, red deer, and wild boar) [15, 28, 29] in the PNCVDA. By analyzing the spatial distribution of these large mammals and their wild prey, we can shed light on the complex ecological relationships that underpin the current and future viability of large carnivores in Mediterranean mountain ecosystems and inform the development of management approaches for their conservation, sustainable forest use and human–wildlife coexistence.

## Methods

### Study area

The study was performed in the Cilento, Vallo di Diano e Alburni National Park (PNCVDA, 40°12′ N–15°12′ E, Southern Italy), covering over 1,800 km^2^, encompassing mountains, coastline, forests, and rural villages (Fig. 1b). The PNCVDA represents a significant biodiversity hotspot characterized by a high environmental and climatic heterogeneity. Its flora comprises approximately 1800 native and spontaneous plant species, distributed across 14 habitats recognized as of interest to the European Community. This diversity of habitats supports a rich and varied fauna, including 67 animal species with protected status, making the protected area a critical site for wildlife conservation in the Mediterranean basin. The climate is typically Mediterranean in the coastal areas and more temperate in the inner lands up to 1,898 m.a.s.l. (Mount Cervati).

### Occurrence data collection

To assess the distribution of the wolf and its potential prey and using multiple methods:

- collection of direct and indirect (i.e., footprints, excrement, barking of trees, shrub browsing, tracks) presence information, by diurnal and nocturnal surveys, with walking transects and spotlight method, respectively [26] (from March 2023 to May 2023 and March 2024 to April 2024).
- Camera trapping (specifically in the core area of the wolf) was conducted over a full year (March 2023–April 2024), encompassing all seasons.
- Roadkill reports (collected over a full year from March 2023 to April 2024).
- Information from citizen science (collected over a full year from March 2023 to April 2024).

For optimal position of cameras, we selected areas in the National Park where the greatest quantity of signs of presence ascribable to the wolf and to the wild ungulates were found. We used the Browning Recon Force 4 K digital cameras with passive infrared sensor (PIR) and LED flash, set in video mode with a duration of 60 s and refractory period varying from one to ten minutes. PIR sensitivity was regulated from normal to low in locations depending from the disturbance of surrounding vegetation. The devices were installed on the tree trunks, approximately 1 m above the ground and were left active all day.

Analyses of the video trapping data consisted in evaluating every sign allowing to distinguish individuals, such as the horns shape, marks of predation or fighting, peculiarities of the coat color.

Even roadkill data improves knowledge about the presence, distribution, dispersal and foraging mode of the species [31]. The wolf roadkill data were provided by our filed census and the Istituto Zooprofilattico Sperimentale del Mezzogiorno and Carabinieri Forestali dello Stato working in the PNCVDA.

Data collection also incorporated citizen science components, increasingly recognized as a valuable resource for improving species distribution models [32–34]. In particular, volunteers receiving specific training on basic biology and monitoring protocols for target species, were provided with a field sheet to record information such as coordinates, time, date, type of observation and any possible audio-photographic documentation.

Data from interviews with local farmers, hunters, and shepherds, and information uploaded by the public to online platforms (www.iNaturalist.org) were critically evaluated before use, following approach proposed by [35]. In particular, all the information used as records had been previously validated by experts; uncertain reports (e.g., data provided as hypothesis, geographically imprecise, blurry photos,) were excluded from the analysis.

All presence data (790 occurrence points in total: wolf, 280; wild boar, 329; red deer, 55; roe deer, 126) were georeferenced according to coordinate reference system WGS 84/UTM zone 33 N. We addressed the spatial autocorrelation of occurrence points by using spatial thinning in R 4.2.1 [36, 37] to ensure that each raster cell contains only one occurrence point and remove duplicates (occurrences with the same coordinates and date). Finally, we visually inspected the distribution of occurrences from each source to ensure that their integration increased geographic representativeness without overemphasizing regions with high observer density.

### Potential distribution analysis

To evaluate potential distribution of the target species, presence data were employed in spatial elaboration by using Maximum Entropy Distribution Model (MaxEnt, [38]) in MaxEnt 3.4.1 software (http://biodiversityinformatics.amnh.org/open_source/maxent/). This methods is considered one of the best among many other Spatial Distribution Modelling (SDM) approach [39] in conservation biology [40–42] since it requires only presence data and environmental variables, exploiting continuous and categorical data at a single point in time [38, 43, 44]. Furthermore, it is less sensitive compared to other approaches to the number of locations needed to provide an accurate model [45].

We selected 12 environmental variables (EVs) (Table 1): aspect, elevation, slope, distance form agricultural meadows, broadleaf forests, conifer forests, grasslands, mixed woods, scrublands, tree plantations, urban settlement, and waterways. All EVs are descriptive of the species requirements according to their ecology inferred from previous studies on the habitat selection of wild ungulates and wolf. For the latter, we included also potential distribution of wild boar, red deer and roe deer as variables [13, 19, 46–50].

**Table 1:** Estimates of percent relative contribution and percent permutation importance (in brackets) of the environmental and landscape variables to the Maxent model for Wolf, Wild boar, Red deer and Roe deer. Dist., distance

Variable	% Relative contribution (and % Permutation importance)			
	Wolf	Wild boar	Red deer	Roe deer
Aspect	0.3 (2.4)	2.0 (1.7)	1.4 (2.6)	0.0 (0.1)
Dist. from agricultural meadows	0.5 (3.9)	50.6 (37.1)	0.7 (0.6)	0.0 (0.0)
Dist. from broadleaf forests	0.7 (0.6)	0.6 (1.1)	0.0 (0.8)	0.2 (2.8)
Dist. from conifer forests	0.5 (0.5)	9.6 (24.5)	0.6 (0.6)	0.1 (2.0)
Elevation	2.0 (17.4)	14.0 (14.6)	5.2 (5.2)	23.3 (51.7)
Dist. from grasslands	0.0 (0.1)	3.2 (3.6)	0.1 (0.0)	2.9 (4.3)
Dist. from mixed woods	0.1 (3.2)	1.0 (4.8)	27.6 (68.3)	3.0 (2.6)
Dist. from scrublands	0.0 (0.4)	1.3 (2.0)	0.1 (0.2)	0.0 (0.0)
Dist. from tree plantations	0.1 (0)	1.0 (1.4)	0.1 (0.3)	0.7 (0.1)
Dist. FROM urban settlements	0.2 (0.3)	13.4 (1.3)	61.2 (18.6)	68.6 (31.7)
Dist. from waterways	0.3 (4.3)	0.1 (0.0)	0.0 (0.0)	1.0 (4.5)
Slope	0.0 (0.7)	3.3 (7.9)	3.3 (5.4)	0.1 (0.2)
Potential distribution red deer	0.9 (20.7)	–	–	–
Potential distribution roe deer	85.9 (0)	–	–	–
Potential distribution wild boar	8.4 (45.4)	–	–	–

Elevation was determined by using Digital Terrain Model (DTM, [51]) resampled at 100 m. Aspect and slope were calculated using the gdal functions “Aspect” and “Slope” in GDAL functions in QGIS3.10 (http://qgis.org/), respectively. All other variables were obtained as categorical vectors from Corine Land Cover 2018 classes (EEA, 2018).

Multicollinearity among variables was evaluated by using Variance Inflation Factor (VIF) [52], applying a threshold of 5. Prior to analyze the data, missing values were removed and a random subset of 10,000 raster cells was extracted to reduce computational load. Variables exceeding the VIF threshold were excluded, and the resulting reduced set of predictors was retained (Supplementary Table S1). Thus, pairwise associations among selected predictors were quantified using Pearson’s correlation coefficient [53] (Supplementary Table S2).

To minimize this bias linked to potential overfitting and reduced interpretability [54–56], we optimized model parameters using the ENMeval package [57], varying the regularization multiplier (RM: 0.5–4, with increments of 0.5 under a fivefold cross-validation scheme [58–60] and testing six feature combination (FC): (L, LQ, H, LQH, LQHP, and LQHPT; L, linear; Q, quadratic; H, hinge; P, product, T, threshold) [61]. The best-performing model was selected based on the Akaike Information Criterion (AICc), with additional consideration of omission rates and Area Under the Curve (AUC) when necessary [62, 63]. Based on the ENMeval results (Supplementary Tables S3–S6), we used RM = 4 and FC = L for roe deer and wild boar, while we applied RM = 3.5/FC = LQHP for red deer and RM = 3.0/FC = L for wolf. Logistic output, 10,000 background points, 1,000 maximum iterations, and removal of duplicate coordinates.

Spatial data processing and rasterization were performed in R using sp [64] and sf [65]. Kernel density estimation (KDE) was computed with spatstat.geom [66] using the number of individuals per observation as weights and a smoothing bandwidth (sigma) of 3 km. Density raster’s were generated with raster [67] and normalized to 0–1 to produce a bias layer for MaxEnt. Contour lines were extracted using terra [67] and the normalized raster was used in MaxEnt to account for spatial sampling bias in background point selection.

The MaxEnt output format was set to Cloglog, with 10,000 background points, a maximum of 500 iterations, a convergence threshold of 0.00001, and default prevalence set to 0.5. Duplicate coordinates were removed prior to analysis. Model performance was evaluated using k-fold cross-validation, where the occurrence data were randomly split into equally sized subsets ("folds"). Models were trained by omitting one fold at a time, and the omitted fold was then used for evaluation [68].

Predictive performance was evaluated using the threshold-independent test of model using the AUC (area under the curve) for a ROC (receiver operating characteristic) plot [69, 70], through five‐fold cross‐validation. The AUC value ranges from 0 to 1, and the higher the AUC, the better the model's reliability. We complemented AUC with Maximum Training Sensitivity plus Specificity (MTSS) threshold [71, 72], providing a more balanced and ecologically meaningful evaluation of model performance [61, 71, 73, 74]. Thus, the MaxEnt output was converted to binary form (1 = suitable and 0 = unsuitable), and values greater than 0.95 were considered to have high/maximum environmental suitability.

The environmental variables contributing the most to the model were assessed by percent contribution and percent permutation importance.

The potential distribution maps was obtained converting habitat suitable area in discrete maps using ‘‘10th percentile training presence’’ [38] and ‘‘maximum training sensitivity plus specificity’’ as thresholds [71]. The total area included within these thresholds was used as suitable habitat area and constituted the potential distribution in subsequent analyses. The continuous output format was converted to binary form (1 = suitable and 0 = unsuitable) using the maximum training sensitivity plus specificity threshold [71, 72]. The values ≥ 0.90 were considered to have maximum environmental suitability.

Jackknife tests in the MaxEnt were used to evaluate the relative importance of each variable in predicting the observed distribution. Therefore, the response curves for each variables were generated to illustrate relationship of variables with the modelled probability of occurrence of the species.

### Estimate of Wolf population’s density

**Fig. 2:**
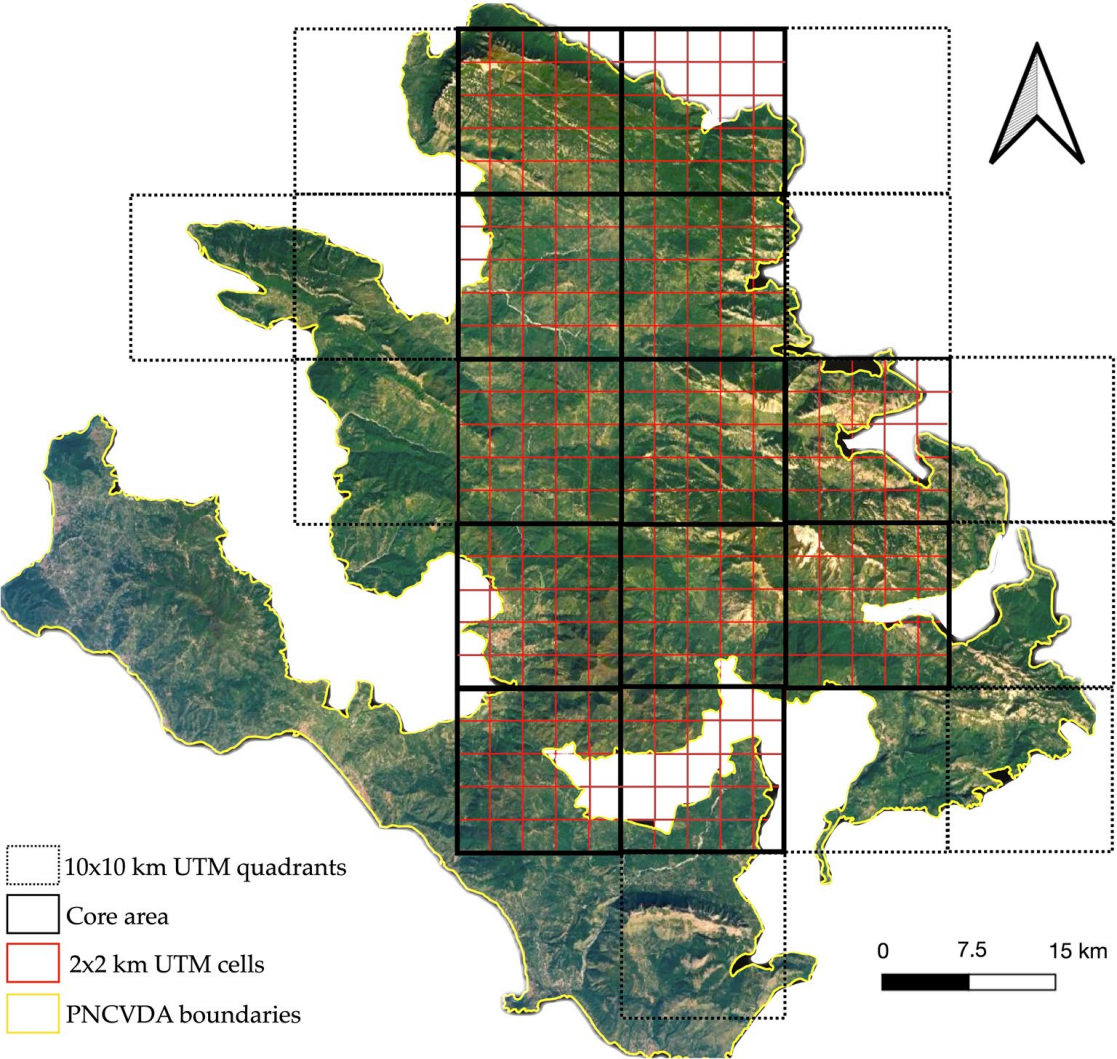
Diagram illustrating how camera trap locations are selected. Sampling cells of 2 × 2 km (red squares) included in the 10 × 10 km quadrants (bold black square) located in core area to evaluate mean density of wolf

To estimate density of wolf in National Park, we used camera trapping data in core-area of the species [4, 13]. Twelve quadrants 10 × 10 km wide include the wolf core area characterized by a different type of habitat. In each of these quadrants, 4 camera traps were placed randomly following a grid of 2 × 2 km cells and considering a feasibly reachable locations (Fig. 2).

To calculate density, we have applied the Random Encounter Model (REM) [75] in R environment [36] and ‘‘camtools’’ functions [76], following equation: D = *y*/ *t*⋅*v*⋅π⋅*r*^*2*^⋅*θ*.

D is the density of the species (individual/km^2^), y/t is the trapping rate, v represents the animal movement speed), r is the radius of the detection zone, and θ refers to the angle of the detection zone (0.77 radians for our camera devices).

Considering uncertainty in movement and detection parameters, and to provide a more robust wolf population density estimate, we applied a parametric bootstrapping approach in R environment [36]. We performed 10,000 simulations, and values for *v* and *r* were randomly drawn from normal distributions based on previous information. In particular, we assumed a mean daily movement of wolf of 16 km/day with a standard deviation of 6 km [77–82], and a mean detection distance of 20 m (± 2 m). Simulations producing non-positive values of *v* and *r* were excluded. For each iteration, we recalculated D, resulting in a distribution of density estimates from which we derived the mean density and the 95% confidence interval, using the 2.5th and 97.5th percentiles.

To estimate the wolf’s population size in the protected area based on density data, we first assumed that only part of the landscape provides suitable habitat for the species. Therefore, density values were applied only to the areas classified as highly suitable for wolf (≥ 0.9).

### Spatial correlation between wolf and its prey

To evaluate spatial correlation between wolf and its prey, pairwise Euclidean distance matrices were computed separately for each species using the dist() function on their spatial coordinates in R environment [36]. To assess the degree of spatial congruence between two species, we performed the Mantel test using the mantel() function from the vegan package, with 999 free permutations to assess significance [83]. To further assess the robustness of the correlation, the distribution of the Mantel statistic under the null hypothesis was also examined. Significant correlations were defined based on a threshold of *p* < 0.05.

### Spatial overlap and correlation analysis according to predicted distributions of wolf and its prey

To identify degrees of spatial congruence or divergence, we evaluate the spatial overlap between predicted distributions of wolf and its prey used Schoener’s D statistic [84], in R using the “terra” package [67].

A correlation analysis was performed also between the potential distribution of wolf and its prey, starting from environmental raster layers (.tiff) of potential distribution of the species. Firstly, the rasters were verified to have consistent resolution and spatial extent using compareGeom() in R environment [36]. When discrepancies were found, the rasters were resampled to match the common template with bilinear interpolation using (resample(), method = “bilinear”). All resampled rasters were subsequently stacked into a multilayer raster object using rast() from the “terra” package [67]. The raster values were converted into a data frame, excluding missing values, to ensure statistical robustness. Thus, a pairwise correlation analysis was performed on the extracted raster values using Paerson’s correlation [85] to evaluate linear relationships among the environmental variables. In addition, Spearman’s rank correlation coefficient [86] was used considering it is more robust to non-linear and non-normal relationships than Pearson’s method [87]. In Spearman’s correlation analysis, to reduce the influence of spatial autocorrelation, rows where all species showed zero values were excluded. A stratified random sampling procedure was then applied, limiting the dataset to a maximum of 5,000 observations, with a fixed seed to ensure reproducibility [88]. Furthermore, to control the false discovery rate due to multiple comparisons, we applied the Benjamini–Hochberg correction [89] using the p.adjust() function. Finally, to assess the statistical significance of the observed correlations, pairwise correlation tests were conducted using the cor.test() function across all variable combinations. Significant correlations were defined based on a threshold of *p* < 0.05.

## Results

### Predator–prey spatial patterns

**Fig. 3:**
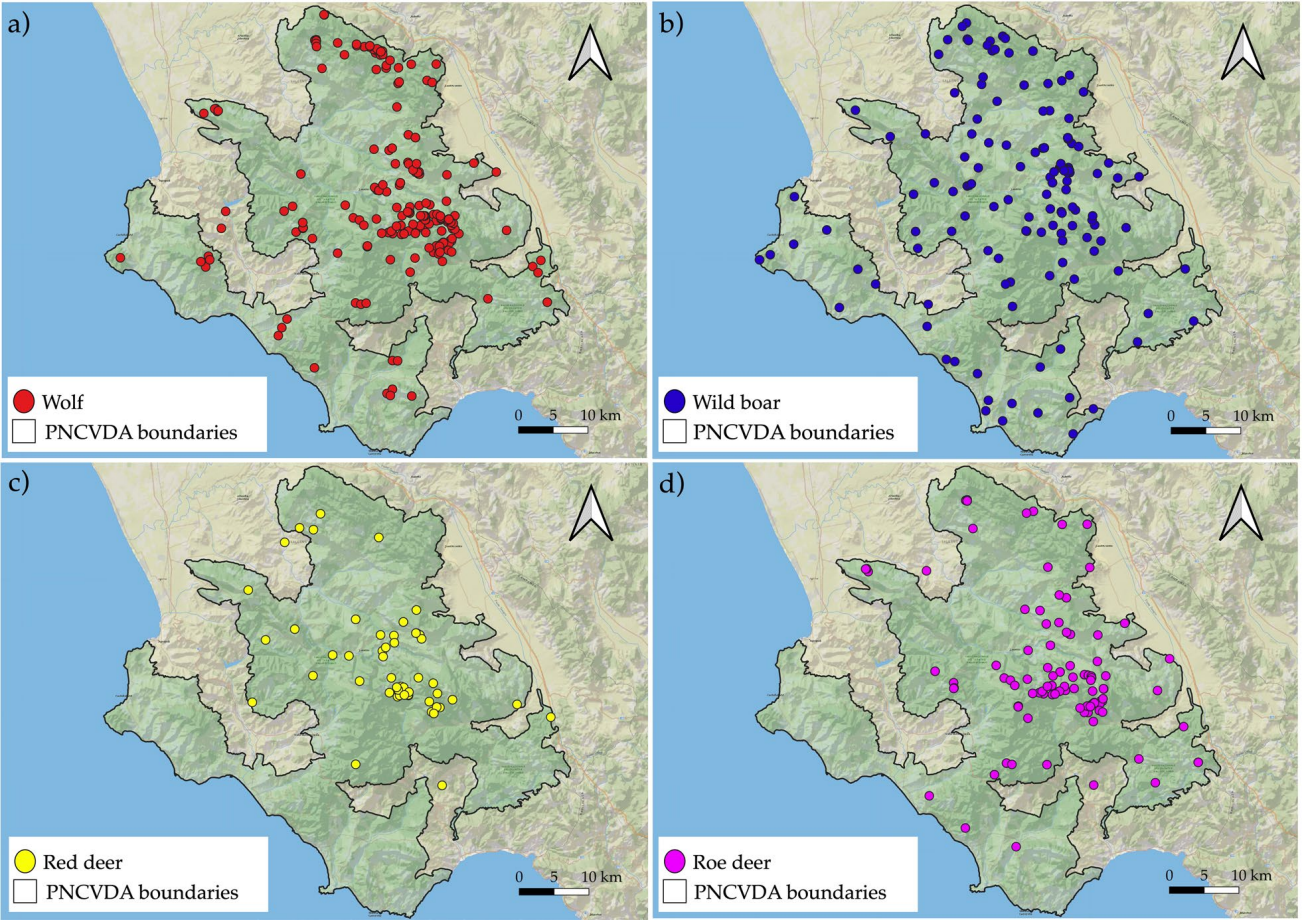
Prey-predator distribution. Distribution map for **a** Wolf, **b** Wild boar, **c** Red deer and **d** Roe deer in Cilento, Vallo di Diano e Alburni National Park, based on verified presence data. Each colored spot indicates a record

The wolf population is widely detectable throughout the Park's territory (Fig. 3a) and the most suitable area exhibits an environmental carrying capacity of 1.2 ind/km^2^, with a 95% confidence interval ranging from 0.41 to 2.99 ind/km^2^, according to Random Encountered Model. This interval could reflect natural variability in wolf movement patterns and the effective detection area of the cameras.

The area of highest suitability for the species (78.95 km^2^; HS ≥ 0.9) could potentially support up to 95 wolves. Despite partial isolation, this population maintains connectivity with the southern Apennine population in at least two areas: the Vallo di Diano and the Piana del Sele (Fig. 1).

Among the prey considered in this study, the wild boar is clearly the most abundant and widely distributed across the entire National Park (Fig. 3b), however, its encounter probability may be influenced by the monitoring methodology.

Both red deer and roe deer are also broadly distributed, although they exhibit a slightly more localized spatial pattern (Fig. 3c, d). In particular, red deer are concentrated in the central-northern part of the Park, avoiding the coastal area. This pattern is similar for the roe deer, which, however, also extends into the southern portion of the protected area.

### Predicted potential distribution

**Fig. 4:**
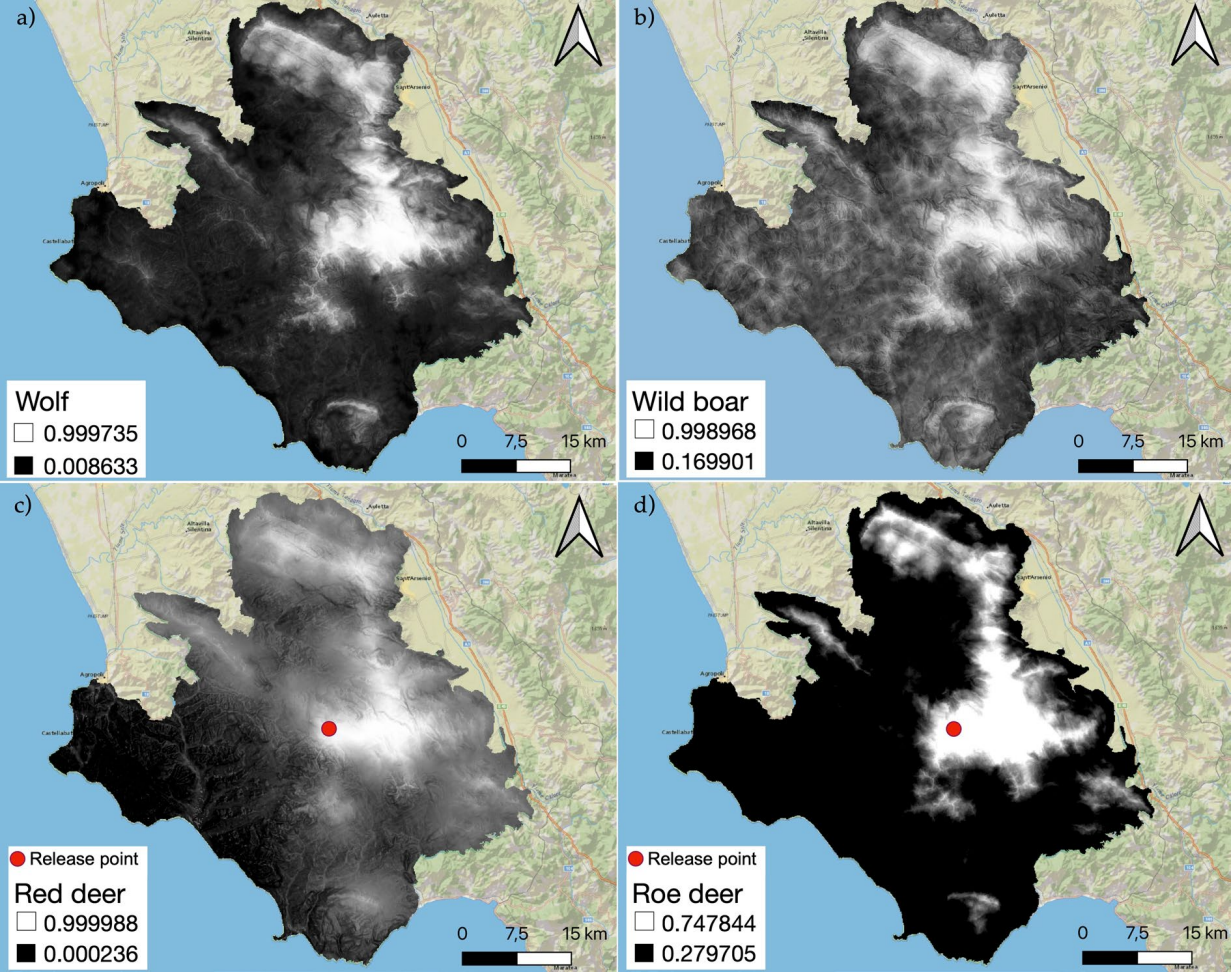
Prey-predator predicted distribution**.** Potential distribution map for **a** Wolf, **b** Wild boar, **c** Red deer and **d** Roe deer in Cilento, Vallo di Diano e Alburni National Park. From white to black the habitat suitability for the species progressively decreases

The thinned presence data enabled elaborating a potential distribution model for each of the four species (Fig. 4) using 13 environmental variables for wild ungulates and 15 variables for wolf, according to VIF output (Supplementary Table S2). The wolf potential distribution model is based on 132 occurrences, reaching an accuracy (defined as AUC value) of 0.811 ± 0.0768 for training data, and AUC of 0.796 ± 0.0816 for test data (Supplementary Figures S1a), suggesting a high performativity of the predictive model [90, 91].

For wild boar, the potential distribution was elaborated using 114 occurrences with AUC 0.671 ± 0.1208 and 0.632 ± 0.0974 for training and test data, respectively (Supplementary Figures S3).

The potential distribution of red deer was based on 45 occurrences (AUC for training data: 0.8204 ± 0.0807; AUC for test data: 0.766 ± 0.1722) (Supplementary Figures S5) while the model for roe deer is based on 74 occurrences, showing a value of AUC of 0.8239 ± 0.0909 for training data and of 0.804 ± 0.0965 for test data (Supplementary Figures S7).

Jackknife test for wolf distribution (Supplementary Figure S1b) shows the roe deer as the variable with the highest gain when used in isolation. This test for wild boar (Supplementary Figure S3b) shows that distance from agricultural meadows is the environmental variable with the highest gain while for red deer (Supplementary Figure S5b) and roe deer (Supplementary Figure S7b) it is represented by the Euclidean distance for urban settlement (urb _dist).

The threshold considered for potential distributions was the maximum training sensitivity plus specificity, with pixels values > 0.406 for the wolf, > 0.598 for wild boar, and values > 0.502 for red deer and > 0.394 roe deer.

Analysis of potential distribution maps highlighted a clear preference for the central mountainous area by the wolf (Fig. 4a), and a widespread potential presence by the wild boar (Fig. 4b). Red deer and roe deer predicted arrangement (Fig. 4c, d) are clearly influenced by the release operations occurred in 2006, as pointed out by the hot spot of highest encounter probability corresponding to the release area on Mount Cervati.

The relative contribution and permutation importance of landscape variables varied across species (Table 1 and Supplementary Fig. S2, S4, S6, S8). The wolf showed a strong dependency on the potential distribution of roe deer (85.9%), followed by potential distribution of wild boar (8.4%) and elevation (2.0%). The distance from urban settlements (68.6%) as well as the elevation emerged as the most important variable for roe deer (23.3) (Table 1), with a negative association with human-modified environments and lower altitudes (Supplementary Figures S8).

Red deer potential distribution was also affected by distance from urban settlements (61.2%), distance form mixed wood (27.6%), and elevation (5.2%). Finally, for wild boar, the variable with a contribution of just over 50% was the distance from agricultural meadows (50.6%), followed by elevation (14.0,%), distance from urban settlement (13.4%) and distance from conifers (9.6%) (Table 1).

### Spatial correlation analysis

**Fig. 5:**
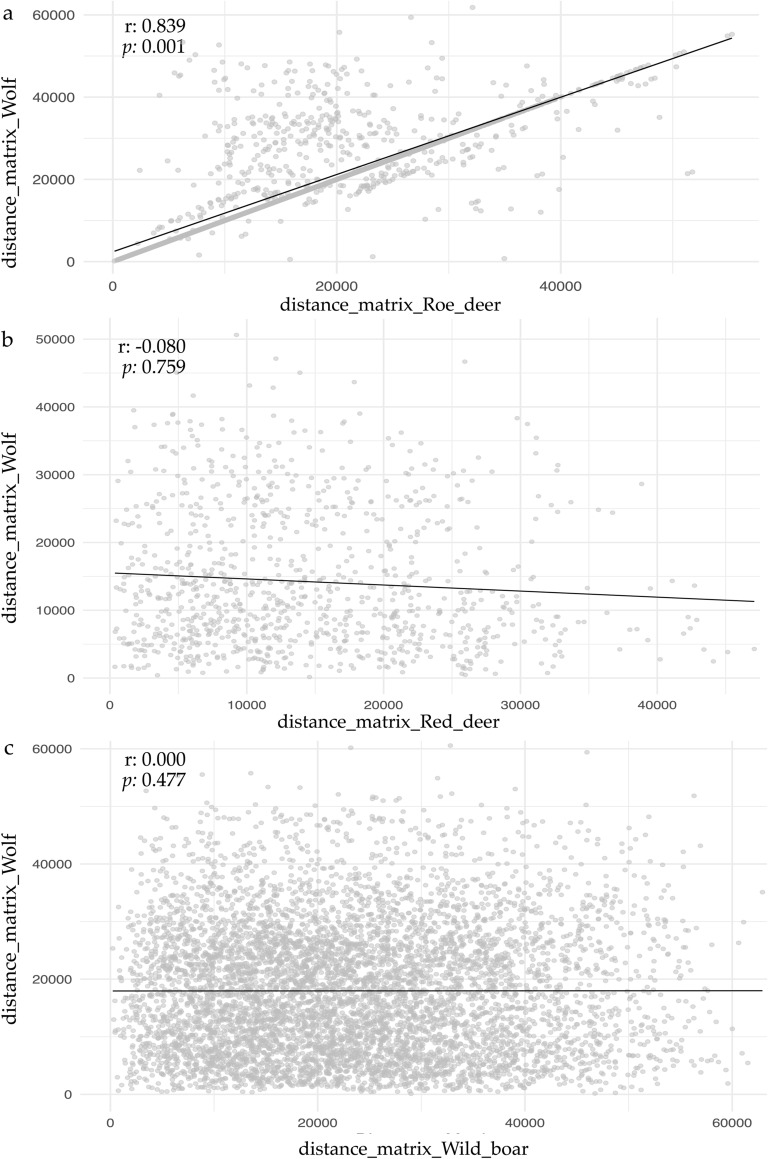
Correlation values for prey-predator distributions. Scatterplot comparing Euclidean distances of **a** Roe deer, **b** Red deer and **c** Wild boar and Wolf. The grey spots indicate individual paired distance observations. The black line represents the linear regression fit (least squares method). Mantel statistics: r, correlation coefficient between the two distance matrices; *p*, significance value

To quantitatively analyze the relationship between the distributions of the predator and its prey, we performed a spatial correlation analysis (Fig. 5). The Mantel test suggests that there is a non-random and significant spatial correlation only between the distributions of the wolf and the roe deer (Mantel statistic, r = 0.8397, *p* = 0.001) (Fig. 5a).

The observed value of r exceeded all upper quantiles of the null distribution (Table 2), indicating that the correlation lies well beyond what would be expected by chance. In contrast, there is no significant evidence that the spatial distribution of the wolf influences that of the red deer (Fig. 5b) or the wild boar (Fig. 5c). In particular, the negative correlation value observed for red deer (r = − 0.0803) suggests a tendency toward spatial segregation, meaning that the two species may avoid one another or occupy different habitats. Furthermore, the observed value fell well within the expected range under the null model (Table 2), suggesting no meaningful correlation. However, the correlations values for red deer *vs.* wolf (*p* = 0.759) and wild boar *vs.* wolf distributions (*p* = 0.477) were not statistically significant(Table 2).

**Table 2:** Mantel statistic to correlate spatial distribution between Wolf and its prey (Roe deer, Red deer and Wild boar)

	Upper quantiles of permutations (null model)				r	*p*
	90%	95%	97.50%	99%		
Roe_deer-Wolf	0.1110	0.1440	0.1176	0.2030	0.8397	0.001*
Red_deer-Wolf	0.1410	0.1840	0.2230	0.2530	− 0.0803	0.759
Wild_boar-Wolf	0.0661	0.0835	0.0982	0.1256	0.0007	0.477

Upper quantiles of permutations (null model) indicate threshold values from the null distribution generated by permutations, used to assess statistical significance. r, correlation coefficient between the two distance matrices; p, significance value. *statistically significant value (*p* < 0.05)

Also Schoener’s D test indicated that the highest spatial potential distribution overlap was observed between the Wolf and Roe deer (D = 0.9041), followed by Red deer (D = 0.8117) and Wild boar (D = 0.7805) (Table 3). These results were consistent with Pearson’s correlation values (0.95, 0.83, and 0.86, Wolf *vs* Roe deer, Red deer and Wild boar, respectively). Similar trends were recognizable for Spearman’s correlation indices (ρ), with the strongest association between Wolf and Roe deer (ρ = 0.907), followed by Red deer (ρ = 0.799) and Red deer (ρ = 0.772). All correlation values were statistically significant (*p* < 0.001) (Table 3).

**Table 3:** Schoener’s D statistic measures potential niche overlap between Wolf and its prey (Roe deer, Red deer and Wild boar)

	Schoener's D	Paerson's correlation	Spearman's correlation
Wolf-Roe_deer	0.9041	0.955*	0.907*
Wolf-Red deer	0.8117	0.834*	0.799*
Wolf-Wild boar	0.7805	0.863*	0.772*

Pairwise correlation coefficients between the potential distributions of Wolf with its prey using parametric Pearson’s and non-parametric Spearman’s (ρ) methods. *statistically significant correlation (*p* < 0.01)

## Discussion

The expansion of the wolf population in the recent decades should be related to several factors, including the abandonment of inland areas, protection of both the landscape integrity and the predator, as well as a renewal of prey availability [92–94].

In this study, we analyze the distribution of the wolf in the PNCVDA, providing updated information on its population estimation and spatial occupancy. Then, we correlate this data with real and potential prey distribution, providing new insights into the spatial association between the wolf and its main wild prey.

The National Park has a mountain system disconnected from the Apennine range along the Italian peninsula. The wolf population in the protected area, which has recently increased, merged with that of the Apennines by crossing the Vallo di Diano (a large, anthropized plain). As a matter of fact, we recorded numerous live and dead wolves on the road, crossing this ecological barrier (*personal observations*).

The wolf in the PNCVDA exhibits a specific habitat selection with a marked preference for mountainous areas, although it goes around with hilly and agricultural environments. In particular, in our analysis the relative weight of altitude is consistent with previous findings reporting the wolf tends to occupy higher, less disturbed habitats in Mediterranean ecosystems [47, 95, 96]. Moreover, interestingly, also urban settlements are among the variables that mostly contribute to the distribution of the wolf, confirming a recent adaptive capacity to occupy human-modified landscapes. However, it should be noted that, in Mediterranean landscapes, human settlements are often distributed across a broad elevational gradient, extending even into mountainous areas at relatively high altitudes. Accordingly, the current landscape of the National Park is characterized not only by lowland settlements located in the plains, but also by mountain villages and rural centers scattered throughout the inner, high-elevation area of the Park.

This study investigating the spatial relationships between predator, prey, and landscape is based on a population of more than a hundred wolves distributed over an area of 1,810 km^2^. This estimate is based on the capacity of the area with maximum suitability (78.95 km^2^; HS ≥ 0.9) to potentially support a density consistent with that calculated by the REM. The number of up to 95 wolves estimated in this 79 km^2^ is affected by intra-population relations and social behavioral. Therefore, these wolves also contribute to the whole population of the entire study area according to the gradient of habitat suitability.

**Fig. 6:**
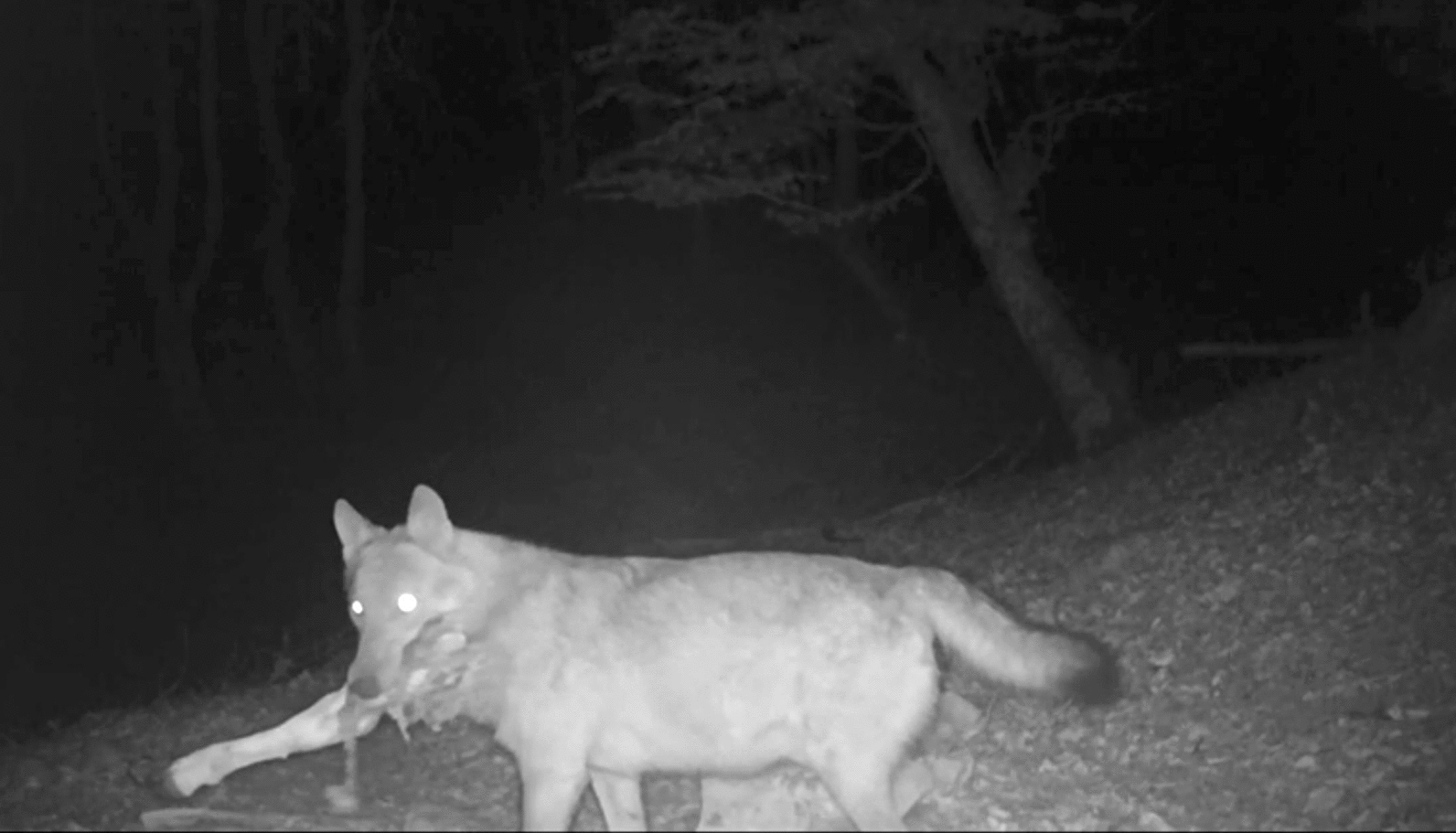
Frame from a camera device showing wolf (*Canis lupus*) carrying the thigh of a roe deer (*Capreolus capreolus*) in the Cilento, Vallo di Diano e Alburni National Park

Besides landscape characteristics, the distribution and density of a predator, such as the wolf, are strongly influenced by the availability of its prey; in particular, our analysis reveals a strong ecological correlation with potential distribution of the roe deer, as reflected in the high relative contribution value (66.7%). The roe deer is also the most spatially associated prey-species with the wolf. As a matter of fact, the Mantel test indicated a significant and non-random spatial correlation between this prey and predator, consistent with Schoener’s D index, Pearson’s as well as Spearman’s correlation analyses. These results could suggest that the wolf and roe deer tend to occur in similar areas, likely due to preference towards this prey [28, 97, 98] or just due to shared environmental preferences. Indeed, roe deer exhibited strong negative association with urban settlements (38.5%) and positive correlation with elevation (29.6%). Theoretically, the dispersal and spatial occupancy of the roe deer, following its reintroduction into the National Park, was influenced by the land structure and habitat quality, and this pattern affected the predator's dispersal routes. This hypothesis does not mean that wild boar and red deer are not prized prey, but it supports that roe deer most influences the dispersal of wolf. Our belief that the roe deer is important in the spatial ecology of the wolf is also due to the numerous screened images with direct predation (Fig. 6).

In our analyses, the potential distributions of red deer and wild boar had notably lower contributions (4.6% and 4.2%, respectively) to the wolf potential distribution.

Wolf shows lower and not significant spatial correlation with the wild boar even though it is considered one of the wolf’s primary and widespread prey (see [27, 28, 47]). According to both presence data and predicted potential distribution analysis, wild boar appears the most abundant ungulate in the protected area, without any particular ecological segregation. This panorama makes it difficult to infer about the possibility that the spatial occupancy of wolf and wild boar could be correlated. Probably, this outcome is influenced by behavioral (i.e. group defense strategies) or ecological (i.e. habitat use) factors with divergence at finer temporal and spatial scales. Thus, further investigation, integrating dietary analysis and fine-scale movement data, would help to formulate a realistic hypothesis to clarify the functional aspects of prey-predator distribution dynamics and spatial patterns.

Red deer appears widely distributed in National Park although it seems to be anchored to the release site (Mount Cervati). It showed limited contribution to wolf potential distribution and the weakest spatial association with the predator. This could be due to specific habitat preferences that do not align with those of the wolf. For example, red deer predicted distribution was strongly influenced by mixed woods (17.7%) which affects potential distribution of wolf only for 0.8%. According to other authors, these findings suggest that spatial proximity between predator and prey is not solely determined by prey abundance, but may also be influenced by habitat selection, ecological compatibility, and prey behavior [99–101]. Moreover, successful attacks on an adult red deer can carry out more likely by large wolf packs. In the protected area, wolves are primarily represented by pairs or dispersing juveniles and rarely from well-structured pack (up to 9 individuals). Large packs have been located only on Mount Cervati and Alburni Mountain areas [13], and recently also on Monte Stella, where nowadays very few deer have been detected. This situation could significantly affect the correlation between prey and predator distributions.

Our study is the first attempt aiming at clarifying spatial wolf-prey relationship in the PNCVDA. However, diet analyses would undoubtedly help to clarify all these observations, providing a more comprehensive understanding of wolf ecology within the Cilento, Vallo di Diano and Alburni National Park. Future researche will address this aspect through genetic and morphological approaches, employing non-invasive methods based on fecal samples to ensure minimal disturbance to wildlife.

We deem that our study on wild prey is crucial to address issues on wolf management in Mediterranean ecosystems. Indeed, the wolf's distribution closely follows the distribution of wild prey, although occasional encounters with livestock may opportunistically lead to predation. Future researche aimed at understanding these spatial relationships can help inform grazing management and land-use planning across the Mediterranean landscape. Moreover, our speculations based on wild prey spatial patterns, also influence the movement routes of young wolves, which during dispersal, tempted by easily available food sources (i.e., human meal scraps), may approach inhabited centers.

## Conclusions

This study provides the first comprehensive assessment of wolf spatial distribution and prey associations in the in one of the largest National Parks in Europe. The findings suggest that the recent expansion of the wolf population is not only promoted by historical factors (i.e. land abandonment, wolf legal protection), but it is also closely linked to prey availability. These results stress the importance of incorporate prey ecology and spatial dynamics into wolf conservation strategies, especially in habitats modified by humans. As the wolf continues to expand, understanding the prey-predator relationships and trophic niche dynamics, considering both wild and domestic prey, is crucial to set successful management planning, trying to mitigate the potential human-wildlife conflicts in peri-urban and agricultural zones.

## Supplementary Information

Additional file1 (DOCX 12458 KB)

Additional file2 (XLSX 53 KB)

## Data Availability

All data generated or analysed during this study are included in this published article and its supplementary information files.

## Abbreviations

PNCVDA: Cilento, Vallo di Diano e Alburni National Park
PIR: Passive infrared sensor
AIC: Akaike information criterion
Area under the curve: AUC
DTM: Digital terrain model
EV: Environmental variable
FC: Feature combination
KDE: Kernel density estimation
LED: Light emitting diode
MaxEnt: Maximum entropy distribution model
MTSS: Maximum training sensitivity plus specificity
ROC: Receiver operating characteristic
REM: Random encounter model
RM: Regularization multiplier
SDM: Spatial distribution modelling approach
VIF: Variance inflation factor
