# Supplementary material for: Where the wolf roams: ecological preferences and wild prey association in a changing Mediterranean landscape
Source: Front Zool. 2026 Feb 5;23:7. doi: 10.1186/s12983-026-00598-2 (PMC12973744; doi:10.1186/s12983-026-00598-2)
Supplement: Supplementary file 1 — Additional file1 (DOCX 12458 KB) [file 12983_2026_598_MOESM1_ESM.docx]

**Supplementary Figures**

Where the wolf roams: ecological preferences and wild prey association in a changing Mediterranean landscape

Maria Buglione^a^, Domenico Fulgione^a*^, Tiziano Trasmondo^a^, Benedetta De Francesco^b^, Gabriele de Filippo^c^, Eleonora Rivieccio^a^

^a^Department of Biology, University of Naples Federico II, Via Cupa Nuova Cinthia 26, 80126 Naples, Italy; [maria.buglione@unina.it](mailto:maria.buglione@unina.it) (M.B.); [t.trasmondo@studenti.unina.it](mailto:t.trasmondo@studenti.unina.it) (T.T.); [eleonora.rivieccio@unina.it](mailto:eleonora.rivieccio@unina.it) (E.R.)

^b^Department of Humanities, University of Naples Federico II, Via Porta di Massa 1, 80133 Naples, Italy; [benedetta.defrancesco@unina.it](mailto:benedetta.defrancesco@unina.it) (B.D.)

^c^Istituto di Gestione della Fauna (IGF), Via M da Caravaggio 143, 80126, Naples, Italy; [igf@gestionefauna.com](mailto:gabrieledefilippo@libero.it) (G.D.)

*****Correspondence: fulgione@unina.it (D.F.) https://orcid.org/0000-0002-5121-3298)

**
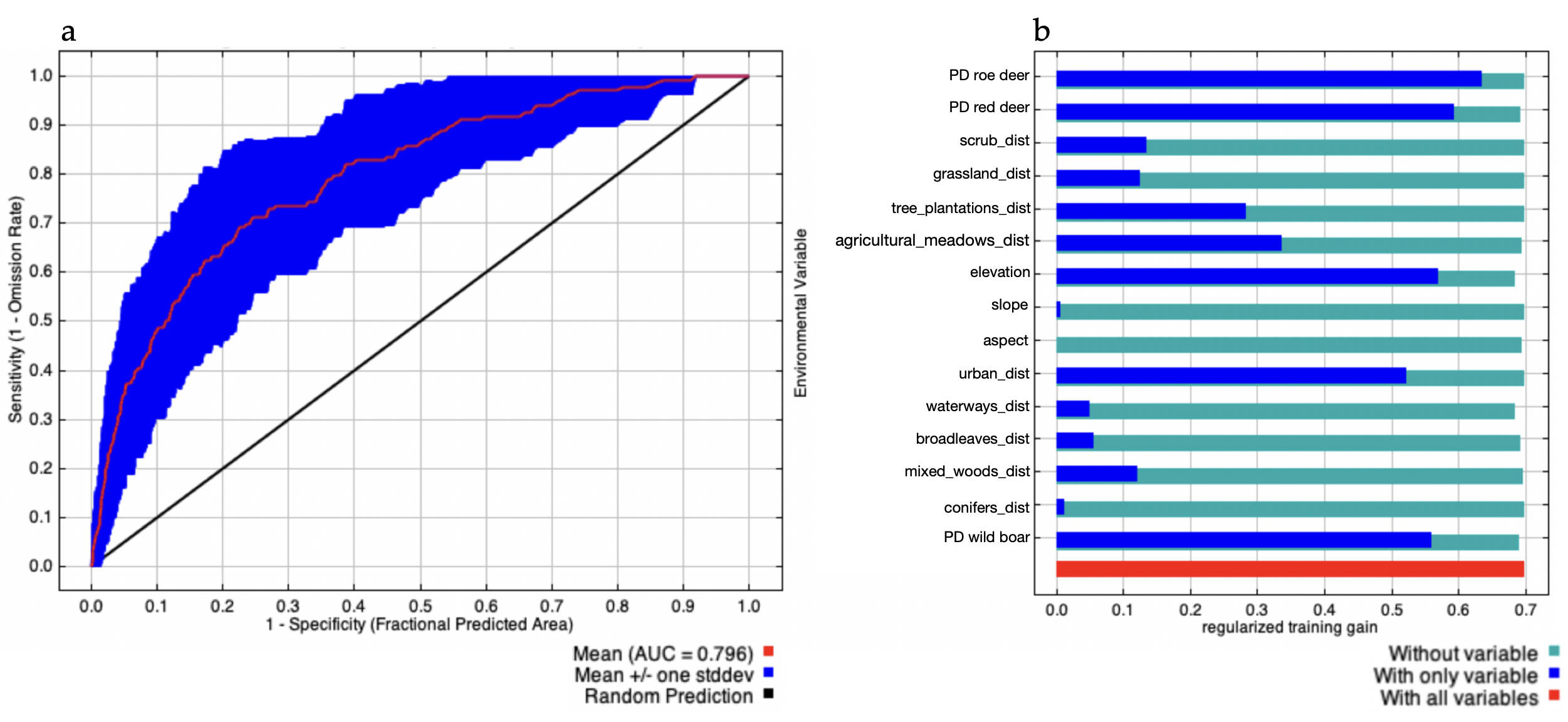
**

**Supplementary Figure S1.** Predictive performance for potential distribution model of Wolf. a) Receiver operating characteristic (ROC) curves for Wolf’s distribution model. AUC, area under the curve. b) Jackknife test of regularized training gain for Wolf. The bar in front of each variable indicates the model's performance when run with only the specified variable (blue) or without it (green). The red bar indicates the model's performance when run with all the variables). PD, potential distribution. _dist, distance from.


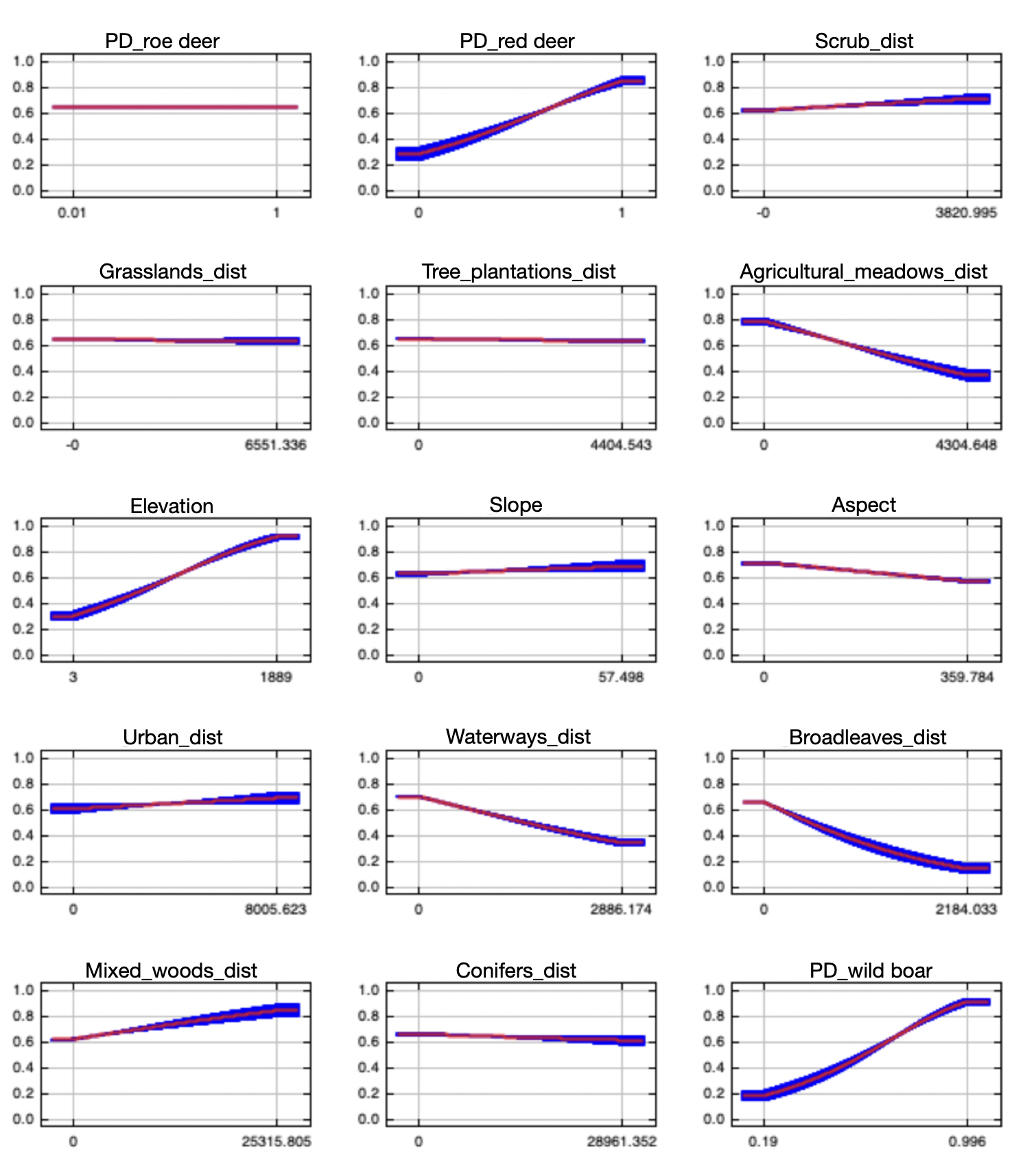


**Supplementary Figure S2.** Response curves probability to key environmental predictors for Wolf. The response curves show the marginal effect of a single environmental variable on the predicted probability of species presence how the predicted probability of presence changes as each environmental variable is varied, keeping all other environmental variables at their average sample value for the Italian hare. The x-axis reports the gradient of the environmental variable; the y-axis represents the predicted probability of presence. Shaded blue bands indicate the 95% confidence intervals derived from model uncertainty. PD, potential distribution. _dist, distance from.

**
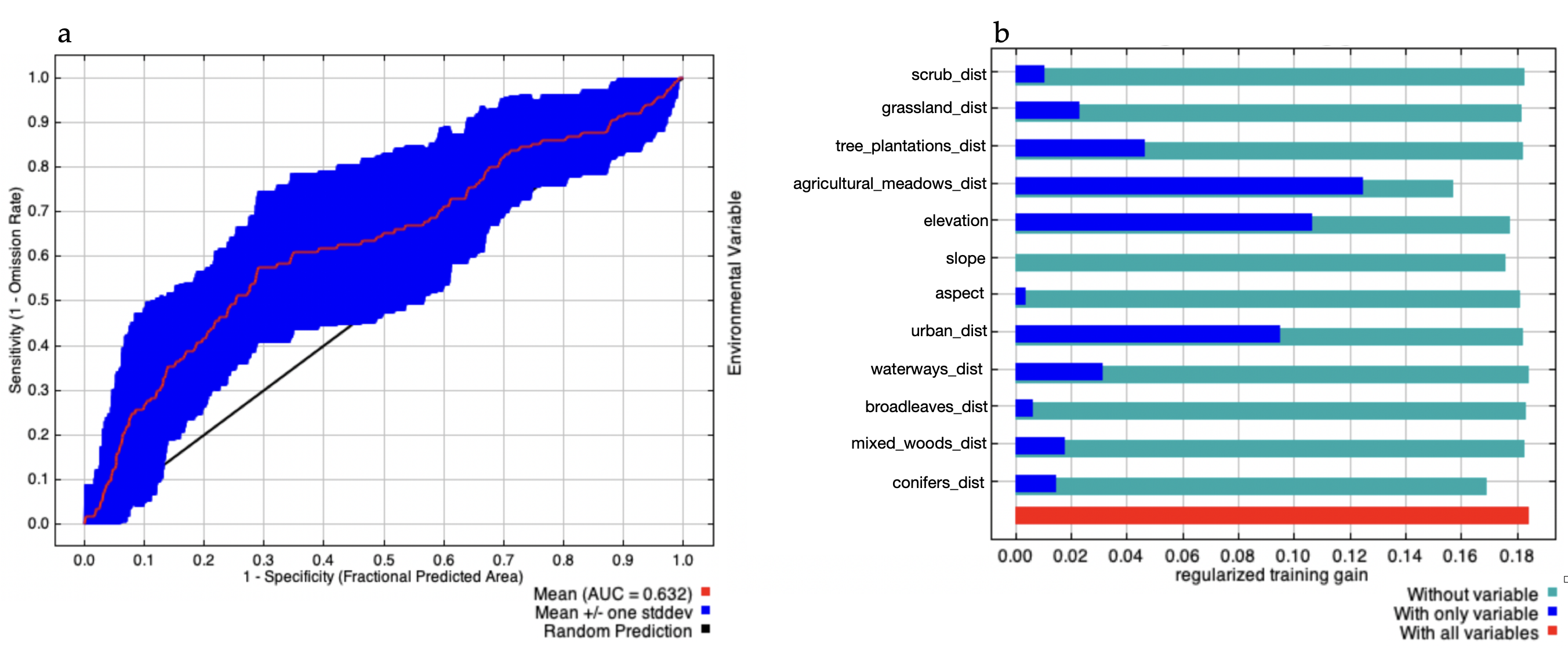
**

**Supplementary Figure S3.** Predictive performance for potential distribution model of Wild boar. a) Receiver operating characteristic (ROC) curves for Wild boar’s distribution model. AUC, area under the curve. b) Jackknife test of regularized training gain for Wolf. The bar in front of each variable indicates the model's performance when run with only the specified variable (blue) or without it (green). The red bar indicates the model's performance when run with all the variables). _dist, distance from.


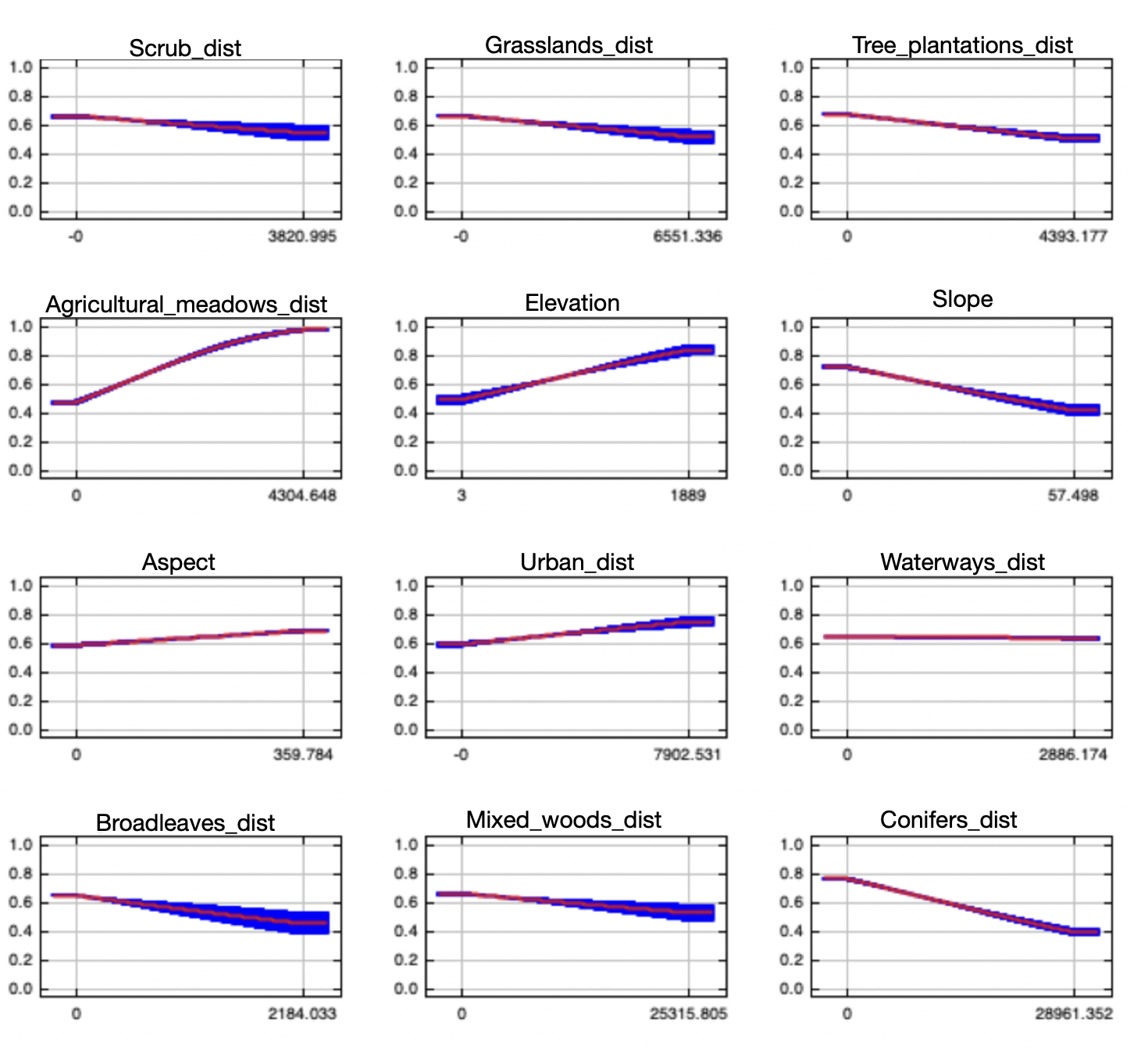


**Supplementary Figure S4.** Response curves probability to key environmental predictors for Wild boar. The response curves show the marginal effect of a single environmental variable on the predicted probability of species presence how the predicted probability of presence changes as each environmental variable is varied, keeping all other environmental variables at their average sample value for the Italian hare. The x-axis reports the gradient of the environmental variable; the y-axis represents the predicted probability of presence. Shaded blue bands indicate the 95% confidence intervals derived from model uncertainty. _dist, distance from.


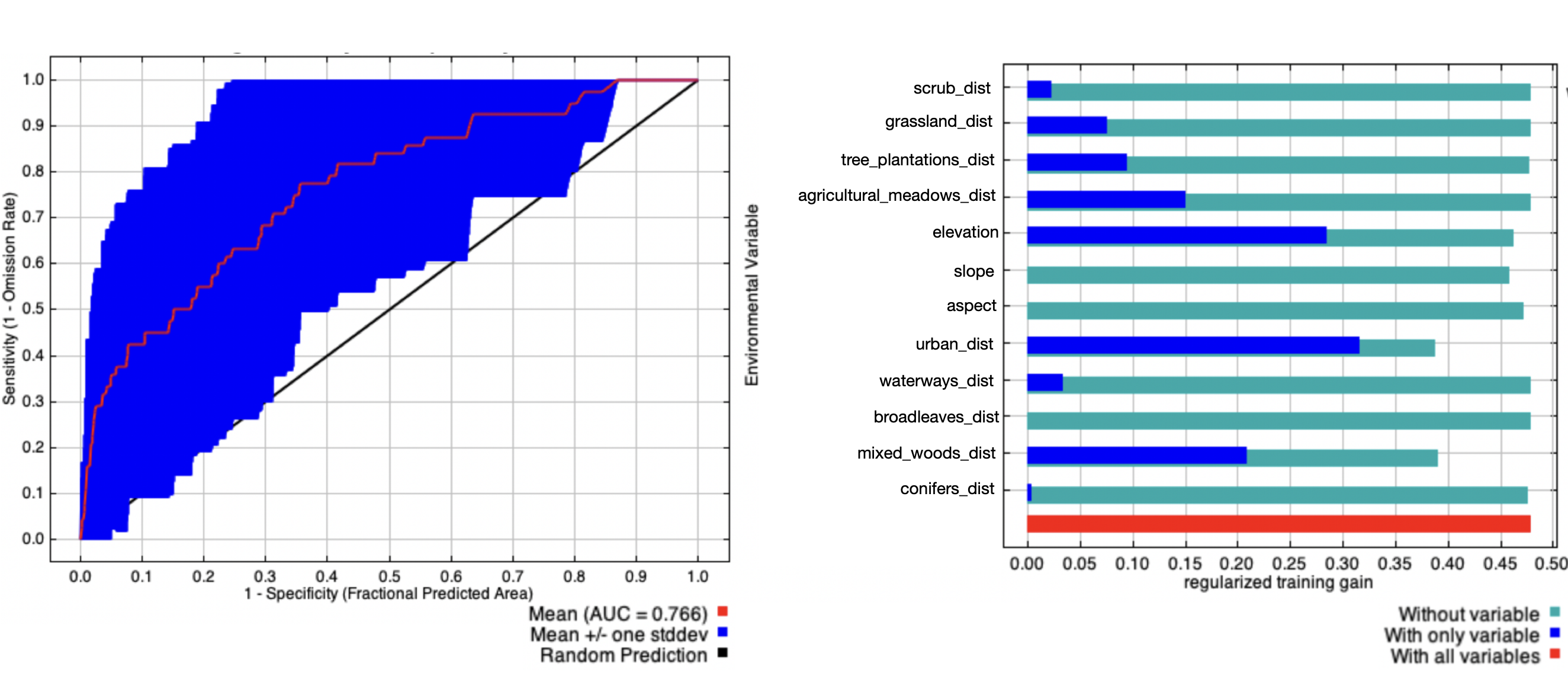


**Supplementary Figure S5.** Predictive performance for potential distribution model of Red deer. a) Receiver operating characteristic (ROC) curves for Red deer’s distribution model. AUC, area under the curve. b) Jackknife test of regularized training gain for Wolf. The bar in front of each variable indicates the model's performance when run with only the specified variable (blue) or without it (green). The red bar indicates the model's performance when run with all the variables). _dist, distance from.

**
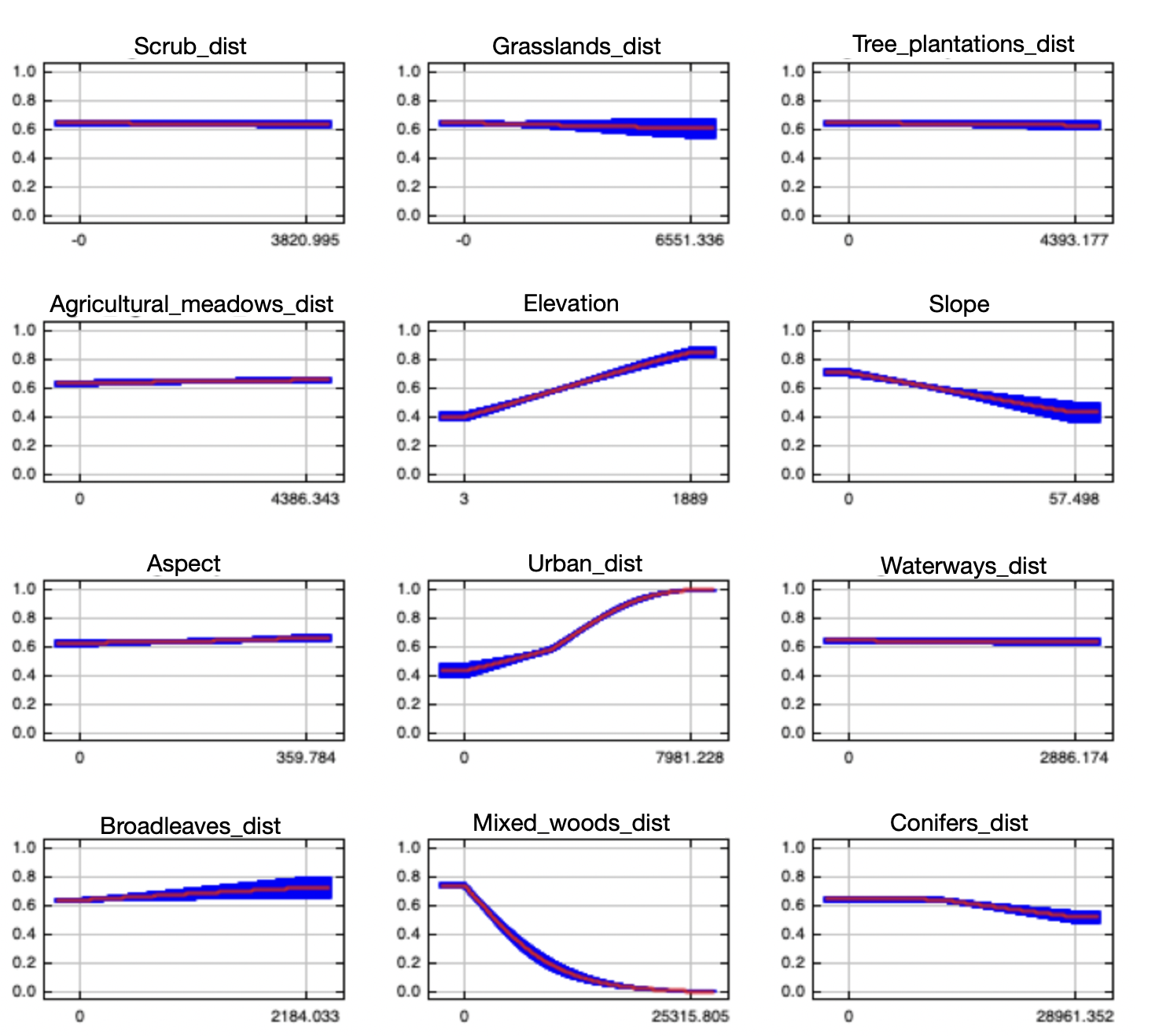
Supplementary Figure S6.** Response curves probability to key environmental predictors for Red deer. The response curves show the marginal effect of a single environmental variable on the predicted probability of species presence how the predicted probability of presence changes as each environmental variable is varied, keeping all other environmental variables at their average sample value for the Italian hare. The x-axis reports the gradient of the environmental variable; the y-axis represents the predicted probability of presence. Shaded blue bands indicate the 95% confidence intervals derived from model uncertainty. _dist, distance from.


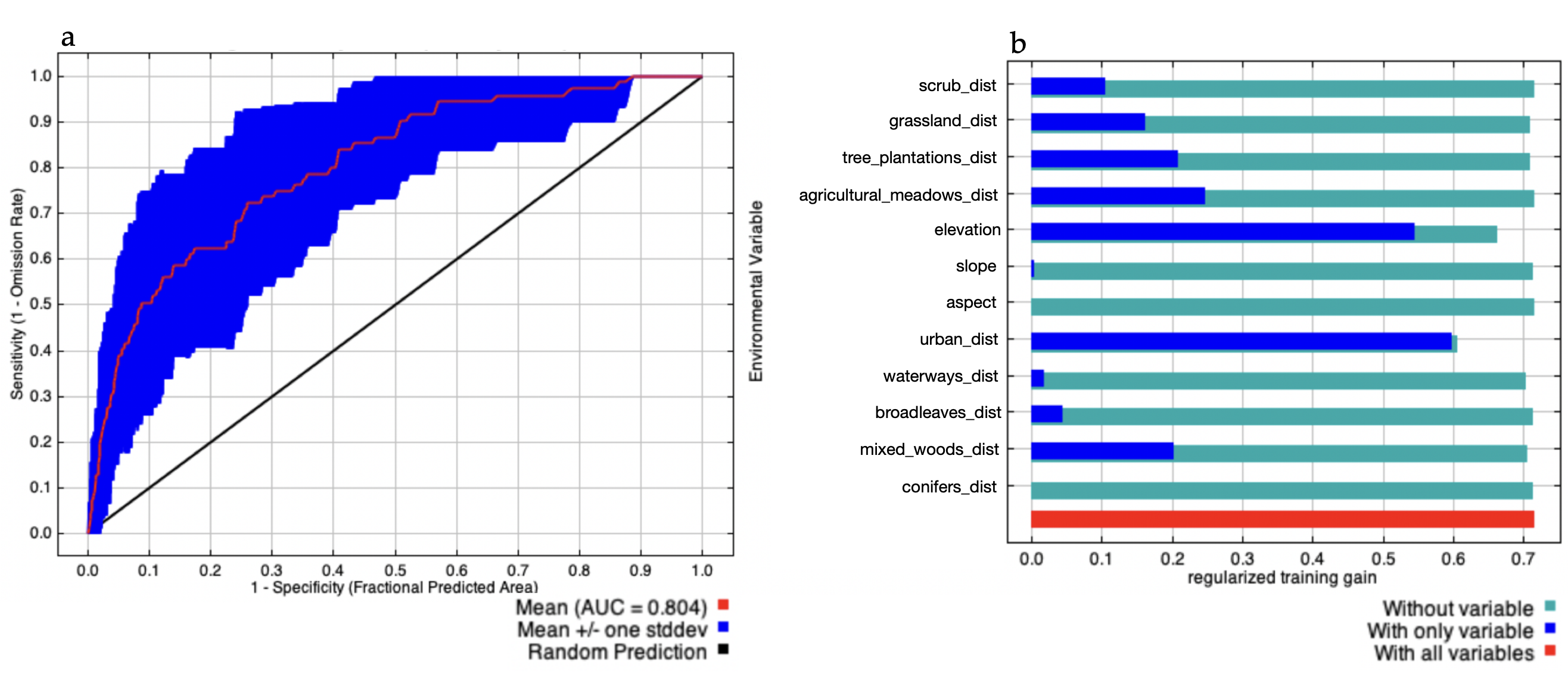


**Supplementary Figure S7.** Predictive performance for potential distribution model of Roe deer. a) Receiver operating characteristic (ROC) curves for Roe deer’s distribution model. AUC, area under the curve. b) Jackknife test of regularized training gain for Wolf. The bar in front of each variable indicates the model's performance when run with only the specified variable (blue) or without it (green). The red bar indicates the model's performance when run with all the variables). _dist, distance from.

**
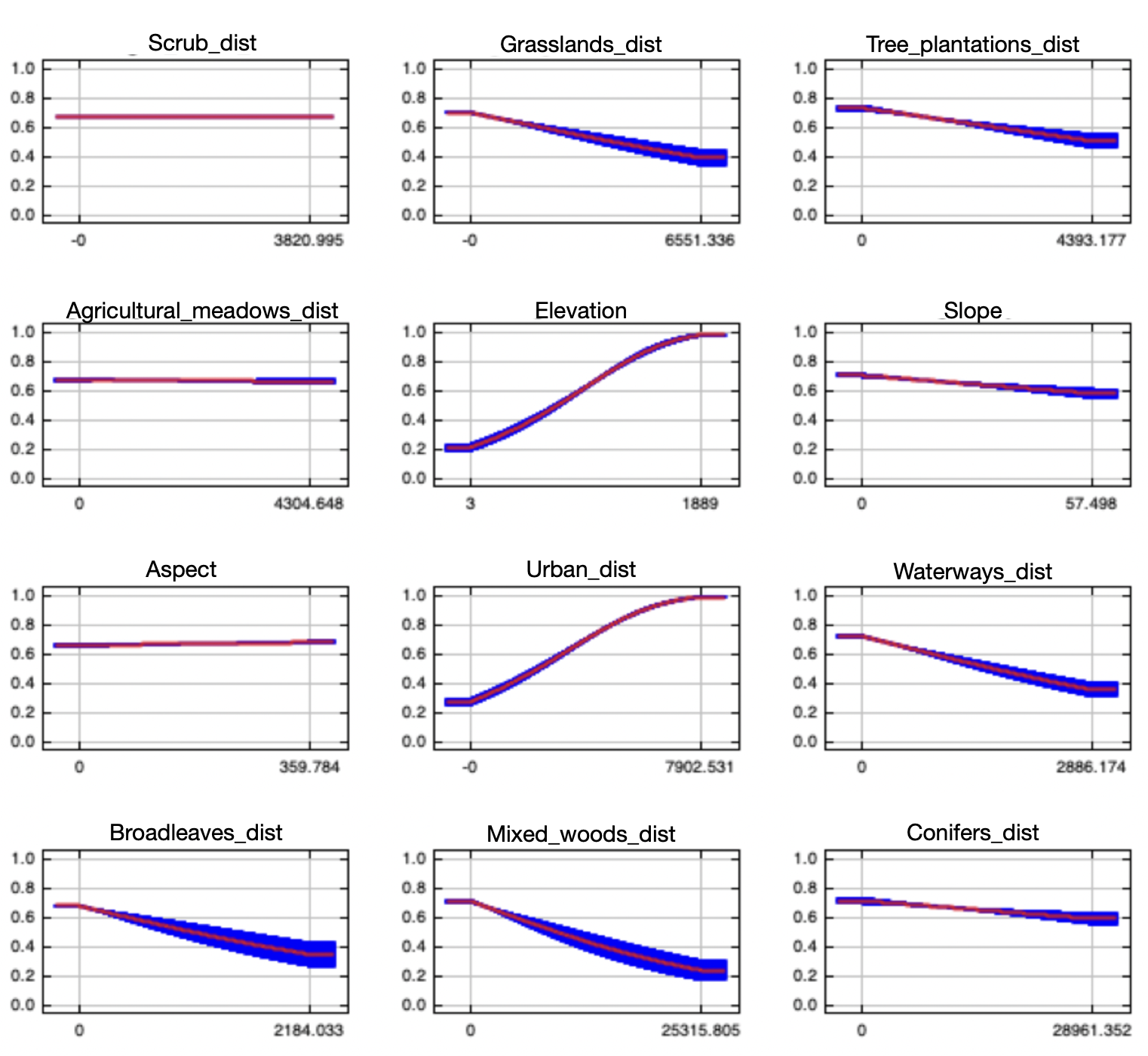
Supplementary Figure S8.** Response curves probability to key environmental predictors for Roe deer. The response curves show the marginal effect of a single environmental variable on the predicted probability of species presence how the predicted probability of presence changes as each environmental variable is varied, keeping all other environmental variables at their average sample value for the Italian hare. The x-axis reports the gradient of the environmental variable; the y-axis represents the predicted probability of presence. Shaded blue bands indicate the 95% confidence intervals derived from model uncertainty. _dist, distance from.
